# Association of *NR1I2* gene polymorphisms and time of progression to AIDS

**DOI:** 10.1590/0074-02760160382

**Published:** 2017-04

**Authors:** Rúbia Marília de Medeiros, Carolina Fialho Menti, Jéssica Louise Benelli, Maria Cristina Cotta Matte, Marineide Gonçalves de Melo, Sabrina Esteves de Matos Almeida, Marilu Fiegenbaum

**Affiliations:** 1 Fundação Estadual de Produção e Pesquisa, Centro de Desenvolvimento Científico e Tecnológicos, Porto Alegre, RS, Brasil; 2 Universidade Federal do Rio Grande do Sul, Programa de Pós-Graduação em Genética e Biologia Molecular, Porto Alegre, RS, Brasil; 3 Universidade Federal de Ciências da Saúde de Porto Alegre, Faculdade de Biomedicina, Porto Alegre, RS, Brasil; 4 Universidade Federal de Ciências da Saúde de Porto Alegre, Programa de Pós-Graduação em Patologia, Porto Alegre, RS, Brasil; 5 Nossa Senhora da Conceição Hospital, Serviço de Doenças Infecciosas, Porto Alegre, RS, Brasil; 6 Universidade Feevale, Novo Hamburgo, Brasil

**Keywords:** AIDS progression, genetic polymorphisms, nuclear receptors, NR1I2

## Abstract

**BACKGROUND:**

The time of progression towards AIDS can vary greatly among seropositive patients, and may be associated with host genetic variation. The *NR1I2* (PXR) gene, a ligand-activated transcription factor, regulates the transcription immune pathway genes and can therefore be targets of viral replication mechanisms influencing time of progression to AIDS.

**OBJECTIVE:**

To verify the association of single nucleotide polymorphisms (SNPs) rs3814057, rs6785049, rs7643645, and rs2461817 in the *NR1I2* (PXR) gene with progression to AIDS in HIV-1 infected patients.

**METHODS:**

Blood samples were obtained from 96 HIV-1 positive individuals following informed consent. DNA was isolated and genotyped through real time polymerase chain reaction (PCR) for the presence of SNPs in the *NR1I2*. Questionnaires on socio-demographic features and behaviors were answered and time of progression to AIDS was estimated based on medical chart analysis.

**FINDINGS:**

Patients with the GG genotype for rs7643645 were shown to be related with a more rapid disease progression when compared to GA and AA genotypes. This result was maintained by the Multivariate Cox Regression considering sex, ethnicity, and presence of HLA-B*57, HLA-B*27, and *CCR5del32* polymorphisms.

**MAIN CONCLUSIONS:**

Recent studies reported the expression of the nuclear receptors in T-Lymphocytes, suggesting their possible role in the immune response. In addition, nuclear receptors have been shown to inhibit the HIV replication, although no such mechanism has been thoroughly elucidated to date. This is the first time an association between *NR1I2* polymorphism and time of progression to AIDS is reported and supports an apparent relationship between the gene in the immune response and identifies another genetic factor influencing AIDS progression.

The time of progression towards AIDS can vary greatly among seropositive patients, ranging from as little as three to as long as 20 years after infection. Previous studies estimated that 10% of HIV positive patients develop AIDS within two to three years (rapid progressors), and 5-15% will remain AIDS-free after 20 years (slow progressors) (Casado et al. 2010). Several attempts to explain differential progression towards AIDS have been made, and so far multiple genes have been shown to play a role in shaping individual time of progression, affecting susceptibility, transmission, and course of disease, ultimately determining viral set points, rate of decline of CD4+ T-cells, level of viremia and cytotoxic T lymphocytes escape (Kaur & Mehra 2009). The extent to which each of those genes influences AIDS onset is not clear, but it is understood to be the result of multiple small factors combined. Nuclear receptors (NR), a family of transcription factors (TF) which comprise the so called orphan receptors, are among the host factors currently known to have some influence in AIDS progression (Hanley et al. 2004). Hanley et al. (2010) and his team have shown two specific NRs that play an import role in diminishing HIV replication through specific mechanisms in vitro. The *NR1I2* (PXR) gene, ligand-activated TFs, regulate the transcription of a wide range of genes (Xie & Evans 2001, Urquhart et al. 2007), including immune pathways (Martínez et al. 2007) and can therefore be targets of viral replication mechanisms influencing time of progression to AIDS. The *NR1I2* (PXR) is also a key to gene regulation, influencing morphogenesis and differentiation, and it may also play a role in the cell cycle progression (Schote et al. 2007, Zhou 2016). Currently available studies also indicate that PXR regulates CYP3A4 expression (Urquhart et al. 2007), which is involved in the metabolism of antiretrovirals and therefore could be a key predictor of drug responsiveness and toxicity (Schipani et al. 2010, Sinxadi et al. 2015, Cusato et al. 2016). This study aims to investigate an association of polymorphisms in the PXR gene with time of progression to AIDS.

## MATERIALS AND METHODS

*Study sample* - Ninety-six blood samples were obtained from HIV-positive patients following stringent review of 3500 medical charts between 2011-2013 in the hospital located in Porto Alegre city, Southernmost Brazil.

For these patients, it was possible to clearly determine the time of progression for AIDS, considering the middle time between HIV-negative and HIV-positive serology, using the retrospective data of their medical records. It is important to note that this parameter is in accordance with the prevailing Brazilian guidelines for the management of HIV+ patients in the country affective in 2013. The Ministry of Health Guidelines for Antiretroviral Treatment indicates that the initiation of Highly Active Antiretroviral Therapy (HAART) should occur for both symptomatic and asymptomatic HIV-1 patients with CD4+ T-cell counts below 350 cells/mm3 and/or a persistently high viral load (NP 2012).

Data available in the medical records, such as the date of seroconversion, HIV-negative serology date, HIV-positive serology date, date of antiretroviral treatment initiation, and lymphocyte count CD4+, were used to classify patients according to the following criteria: (i) rapid progressors, the period from seroconversion (considering middle time between HIV-negative and HIV-positive serology) to the beginning of treatment for AIDS of up to three years, with at least two consecutive CD4+ counts < 350 cells/mm^3^ before data collection and HAART medical recommendation; (ii) typical progressors, the period from HIV-positive serology (with clinical monitoring) to the treatment for AIDS of over four years, with at least three consecutive CD4+ T lymphocytes counts > 500 cells/mm^3^ before data collection and HAART medical recommendation; (iii) slow progressors, the period from HIV-positive serology exceeding nine years, with CD4+ T lymphocytes counts > 500 cells/mm^3^ and without requiring treatment for AIDS. The analyses were performed using this classification in rapid, chronic, and slow progressors.

Other data concerning the individual features of patients were obtained through a sociodemographic questionnaire. The individuals were classified as of European or African ancestry, according to the phenotypic characteristics of individuals, as judged by the researcher at the time of data collection, and from data about the ethnicity of the parents/grandparents reported by the participants. According to Pena et al. (2011), the Brazil Southernmost Region showed reduced mixing compared to the rest of the country, due to great geographical variances and cultural differences in the country. The issue concerning the skin-color-based classification criteria used in this geographical area of Brazil is well documented and has already been assessed in previous studies (da Silva et al. 2011).

*DNA extraction and genotyping* - Total DNA extraction from blood samples collected was performed through the well-established technique of salting out described by Lahiri and Nurnberger (1991). Genotyping of four single nucleotide polymorphisms (SNPs) in the *NR1I2* gene rs3814057 (A > C; mRNA 3’UTR), rs6785049 (G > A; intronic region), rs7643645 (A > G; intronic region), and rs2461817 (A > C; intronic region), were performed by real time polymerase chain reaction (PCR) and validated by TaqMan assays (Applied Biosystems, California, USA). The SNPs evaluated here were selected using the HapMap tool (www.hapmap.org) with the following annotate TagSNP Picker features: European population (CEU), minimum frequency of the rarest allele of 10% and a determination coefficient (R^2^) equal to 80%. HLA-B*27, and HLA-B*57 and *CCR5del32* genotypes were available for the sample and used to control for known genetic factors involved in the progression to AIDS. The *CCR5del32* (rs333) variant was previously evaluated by PCR and visualised on 3% agarose gel, as described by Chies and Hutz (2003). The presence of HLA-B*27 and HLA-B*57 were previously genotyped according Cesbron-Gautier et al. (2004).

*Statistical analysis* - Categorical variables are described in proportions and continuous variables and are expressed as the mean [standard deviation (SD)] or median [interquartile range (IQR)]. Allelic frequencies were estimated through gene counting. The agreement of genotype frequencies with Hardy-Weinberg equilibrium expectations was tested using Chi-square tests.

Initially, patients enrolled were grouped as rapid, chronic, and slow progressors according to the data obtained from the questionnaire and review of the subjects’ medical charts. Their CD4+ T-cell slope values before HAART were estimated by linear regression with the square root of the CD4+ T-cell count and a non-parametric Mann-Whitney and exact Fisher test were used to characterise the progression of the patients according socio-demographic characteristics of patients. After characterisation of the sample, all variables were tested using Cox regression in a univariate model. Multivariate Cox regression was performed considering a forward stepwise model and all three models with statistical significance are shown on results. Genetic recessive models for *NR1I2* polymorphisms were assumed. All statistical analyses were performed with IBM SPSS Statistics for Windows, version 19.0 (IBM Corp, Armonk, NY) and p < 0.05 was considered to indicate statistical significance.

*Ethics* - The study protocol was approved by the Ethical Committees of the Conceição Hospital Group (Porto Alegre) (approval number 10-213) and Fundação Estadual de Produção e Pesquisa (FEPPS) (approval number 13/2010). Written informed consent was obtained from each subject included in the study.

## RESULTS

*Characteristics of study population* - Table I contains data regarding sample description and comparison of clinical and socio-demographic variables between slow, typical, and rapid progressors. Female sex was slightly more frequently observed among slow and typical progressors than in rapid progressors (p = 0.067). Characterisation of disease progression included significant differences (p < 0.001) in the values of CD4+ T-cells slopes: the number of CD4+ T-cells decreased more abruptly in rapid progressors (median slope -0.988; IQR -2.424 to -0.399) and typical progressors (median slope -1.090; IQR -2.649 to -0.427) than in the slow progressors (median slope -0.473; IQR -0.881 to -0.160). Twenty-three patients in the slow group did not start antiretroviral therapy at the time of study completion (follow-up median = 9.75 years; IQR = 8.25-12.25 years).

**TABLE I:** Socio-demographic and clinical features of study sample

Variable	Total (96)	Type of progression			p value

		Rapid (20)	Typical (40)	Slow (36)	
Time to AIDS (years)	7.0 (1-15)	1.55(1-3)	5.89(4-8)	11.21(9-15)	< 0.001
Slope CD4+ (cell/mm^3^)	-	-0.988 (-2.424; -0.399)	-1.090 (-2.649; -0.427)	-0.473 (-0.881; -0.160)	< 0.001
Female	75.0%	55.0%	80.0%	80.5%	0.067
Age (years)	40.81(10.21)	42.72 (11.06)	40.07 (11.83)	40.52 (8.43)	0.640
Transmission					
Het	76.0%	75.0%	80.0%	72.2%	0.725
MSM	8.3%	15.0%	7.5%	5.5%	0.458
IDU	8.3%	5.0%	5.0%	14.0%	0.312
Others	6.3%	5.0%	5.0%	8.3%	0.808
Ethnicity					
White	58.3%	57.9%	74.3%	45.0%	0.025
Non-white	41.7%	42.1%	25.7%	55.0%	
Co-infection (yes)	38.5%	55.0%	37.1%	31.8%	0.194

Except for age (mean/standard deviation) and time to AIDS (median/minim and maxim), data are shown as a percentage (%). Exposure category: Het = heterosexual; MSM, = men who have sex with men; IDU = injection drug user; others includes vertical transmission and occupational accident. Co-infections include hepatitis C/B and HTLV.

*Association of NR1I2 gene polymorphisms and time of progression to AIDS* - Table II shows the comparison of *NR1I2* SNPs among rapid, typical, and slow progressors. Although no association was detected for any SNP, an association with the rs7643645 GG genotype was observed when the comparison of rapid plus typical versus slow progressors was performed (p = 0.0216). Considering that sex [hazard ratio (HR) = 1.608, confidence interval (CI)95% 1.000-2.587, p = 0.050] and ethnicity (HR = 1.657, CI95% 1.054-2.606, p = 0.029) showed significance in Cox Regression Univariate test (Table III), and that HLA-B*57, HLA-B*27, and *CCR5 del32* polymorphisms are well known factors that affect time to progress to AIDS, a multivariate stepwise Cox regression was performed. In this analysis, three models were generated. In model three (step 3 on Table III) our results evidenced an association of rs7643645 GG genotype to a shorter time of progression (HR = 2.029, CI95% 1.036-3.975, p = 0.039) when compared to GA and AA genotypes (Table III). No other polymorphisms were significantly associated with time of progression towards AIDS.

**TABLE II:** Genotypic distribution of *NRI1I2* single nucleotide polymorphisms (SNPs) among rapid, typical, and slow progressors

SNP	Type of progression			p value

	Rapid	Typical	Slow	
rs2461817				
AA	4 (21.1%)	7 (17.9%)	3 (10.7%)	
AC	10 (52.6%)	19 (48.8%)	15 (53.7%)	0.866
CC	5 (26.3%)	13 (33.3%)	10 (35.6%)	
rs3814057				
AA	7 (35.0%)	16 (40.0%)	15 (51.8%)	
AG	11 (55.0%)	20 (50.0%)	11 (37.9%)	0.781
GG	2 (10.0%)	4 (10.0%)	3 (10.3%)	
rs6785049				
AA	1 (5.0%)	8 (20.5%)	8 (27.6%)	
AG	13 (65.0%)	18 (46.2%)	12 (41.4%)	0.321
GG	6 (30.0%)	13 (33.3%)	9 (31.0%)	
rs7643645*				
AA	8 (40.0%)	14 (35.0%)	17 (58.6%)	
AG	10 (50.0%)	18 (45.0%)	12 (41.4%)	0.077*
GG	2 (10.0%)	8 (20.0%)	0 (0%)	

*: rapid plus typical *versus* slow progressors, p-value = 0.026.

**TABLE III:** Univariate and multivariate Cox proportional hazards regression analysis concerning rs7643645 SNP and risk for progression to AIDS

	Univariate analysis			

	HR	95%CI		p value
Sex (male)	1.608	1.000	2.587	0.050
Ethnicity (white)	1.657	1.054	2.606	0.029
G homozygosis	1.810	0.915	3.789	0.083
CCR5del32	0.792	0.352	1.742	0.588
HLA-B*57/B*27	0.948	0.452	2.189	0.803

	Forward stepwise multivariate analysis			
	HR	95%CI		p value
Step 1*				
Ethnicity (white)	1.631	1.041	2.789	0.037
*Step 2* **				
Sex (male)	1.736	1.053	2.915	0.028
Ethnicity (white)	1.710	1.136	2.803	0.021
*Step 3* ***				
Sex (male)	1.945	1.161	3.258	0.011
Ethnicity (white)	1.710	1.003	2.614	0.049
G homozygosis	2.029	1.036	3.975	0.039

CI: confidence interval; HR: hazard ratio; *: -2 Log likelihood = 576.99, chi-square = 4.01, p-value = 0.055; **: -2 Log likelihood = 572.50, chi-square = 8.89, p-value = 0.017; ***: -2 Log likelihood = 568.50, chi-square = 12.77, p-value = 0.007.

## DISCUSSION

This study evaluated the possible influence of four common SNPs from *NR1I2* gene in the time of progression to AIDS. The major finding of this study was the significant association of the rs7643645 polymorphism, gender, and ethnicity with a shorter time of progression towards AIDS. In the rs7643645 polymorphism, the genotype GG has a major risk to progression for disease.

The characterisation of AIDS progression is diverse in literature (Kaur & Mehra 2009, Casado et al. 2010), and presence of AIDS symptoms, CD4+ T-cell counts and plasma HIV RNA levels already were considered by different authors to classify the AIDS progression (Nakaiama et al. 2002, Okulicz et al. 2009). This study used retrospective CD4+ T-cell counts to assess the progression to AIDS, so initially the patients’ progression was validated by the significant difference in the slope of decline in CD4+ T-cell numbers between rapid progressors (-0.988, steep slope) and slow progressors (-0.473, gentle slope).

The analysis on the socio-demographic features of patients showed a positive association between female gender and slow progression to AIDS (p-value = 0.019). This finding is in agreement with other studies, which found higher CD4+ TL counts in females than in male HIV-patients (Jarrin et al. 2008). However, a recent study by Thorsteinsson et al. (2012) found no association between sex and onset of AIDS. It is important to also consider the influence of the patient’s selection mode to reach our results. Patients enrolled were those who voluntarily sought health care, which can constitute a bias, since usually in Brazil more women look for health care services than men (Ribeiro et al. 2006) and our collection site (Conceição Hospital Group) has specific health service for pregnant women. Moreover, a positive association between non-white individuals and slow progression to AIDS was also encountered (p-value = 0.053). Okulicz et al. (2009) showed an increased frequency of African-American individuals among long-term non-progressors in a USA cohort. Further, an earlier study of Anastos et al. (2000) had observed an association between slower decline in CD4+ TL and non-white individuals. Despite of the small sample size investigated due the limited data about progression to AIDS and the ethnic composition of Brazilian population, the results show that investigations including populations of different ancestries and geographic regions are important to understand how genetic background can influence immune responses and modulate HIV infection and progression. HIV affects the entire immune system of the patient, so the pathways and genes studied are those associated with the immune system. However, it is increasingly recognised that gene regulation should be mediated, for example, for nuclear transcription factors such as PXR family receptors encoded by *NR1I2*. These receptors form a network, which influences the expression of many other genes. These interactions are complex and may be simulated using function prediction analysis tools made with an online tool (available at http://snpinfo.niehs.nih.gov). The interactions of *NR1I2* involve tumor suppressor genes, cell cycle regulators and differentiation, inflammatory response promoters, genes involved in production of blood cells and the response of cells to retroviral infection. A recent study demonstrated that PXR expression on peripheral blood cells is different in tissue expression, and this variable in combination with other markers can modulate the immune response of human body to the tumor cells. Following this reasoning, considering that the receptor PXR is able to modulate the recognition of abnormal cells, it is questionable and important to study its real relevance in cells infected with HIV (Kong et al. 2016).

The findings in this study regarding the association of allele G homozygosis in rs7643645 and a rapid progression to AIDS are in agreement with Schote et al. (2007), who suggested an apparent role for PXR in immune response. In that study, it was observed that PXRs, among others NRs, are expressed in CD4+ TL, CD8+ TL, CD14+ TL and CD19+ TL, which suggests their function influences expression of molecules involved in immune mechanisms. The mechanism through which PXR correlates to AIDS progression is not all unraveled to date. However, it is known that it is related to the metabolism of the drugs used to treat AIDS patients, given its role in Phase I and II metabolism of xenobiotics and also being suppressed by some drugs. These studies relate the amount of antiretroviral drugs available in the plasma of patients with polymorphisms in *NR1I2* and *NR1I3*, and consequently the resistance to drugs and prognosis of these patients (Healan-Greenberg et al. 2008, Svärd et al. 2010).

It is known, though, that metabolism and inflammation are related mechanisms. In the presence of cytokines and of a specific types of inflammation (acute phase response by lipopolysaccharide), for instance, a decreased expression of PXR and of its target genes is seen (Moore et al. 2000). It has been suggested that a relationship between PXR and NFkappa-beta functions exists, with NF-kb increased action in PXR null mice as well as a decrease of PXR function with inhibition of NF-kbs, demonstrating a potential for immunosuppressive activity of PXR’s ligands (Wahli 2008). This leads to the belief of a possible influence of this NR in the course of immunosuppressive diseases, such as AIDS, as we have found.

Our study presents some limitations and the results must be interpreted considering some issues: (i) we analyse a small sample of HIV+ patients due the limited data about progression to AIDS; (ii) we genotype a small number of polymorphisms and other *NR1I2* SNPs could be associated with the progression to AIDS; (iii) our data do not represent the actual distribution of slow, typical and rapid progressors in HIV + patients, since a high frequency of slow progressors is observed in the sample; (iv) a group of patients who had not started antiretroviral therapy at the time of inclusion in the study was classified as slow progressor. This group was not fully characterised and potentially includes LTNPs patients (long-term non-progressors patients). Despite this, this is the first time that a *NR1I2* gene polymorphism was found to be related to time of progression to AIDS and even though our sample was not an extensive representative, it can be hypothesized that the absence of the rs7643645 SNP in homozygosis (presence of the A allele) is associated with a delayed progression towards AIDS, when also considering ethnicity and gender. This agrees with the widely accepted belief of several different factors influencing AIDS progression and illustrates the importance of considering them all together (McLaren & Fellay 2015). In addition, this finding is in accordance to several recent studies that suggest a role of NR in immune mechanisms and HIV infection. The presented results support the evidence and stress the importance of continuation of the search for additional genes’ activities that might be related to the development of this widespread disease. It also claims for the elucidation of mechanisms through which it occurs and investigation of other NRs in the matter.

## References

[B1] Anastos K, Gange SJ, Lau B, Weiser B, Detels R, Giorgi JV (2000). Association of race and gender with HIV-1 RNA levels and immunologic progression. J Acquir Immune Defic Syndr.

[B3] Casado C, Colombo S, Rauch A, Martínez R, Günthard HF, Garcia S (2010). Host and viral genetic correlates of clinical definitions of HIV-1 disease progression. PLoS ONE.

[B4] Cesbron-Gautier A, Simon P, Achard L, Cury S, Follea G, Bignon JD (2004). Luminex technology for HLA typing by PCR-SSO and identification of HLA antibody specificities. Ann Biol Clin.

[B5] Chies JA, Hutz MH (2003). High frequency of the CCR5delta32 variant among individuals from an admixed Brazilian population with sickle cell anemia. Braz J Med Biol Res.

[B6] Cusato J, Tomasello C, Simiele M, Calcagno A, Bonora S, Marinaro L (2016). Efavirenz pharmacogenetics in a cohort of Italian patients. Int J Antimicrob Agents.

[B7] Silva GK da, Guimarães R, Mattevi VS, Lazzaretti RK, Sprinz E, Kuhmmer R (2011). The role of mannose-binding lectin gene polymorphisms in susceptibility to HIV-1 infection in Southern Brazilian patients. AIDS.

[B8] Hanley TM, Kiefer HLB, Schnitzler AC, Marcello JE, Viglianti GA (2004). Retinoid-dependent restriction of human immunodeficiency virus type 1 replication in monocytes/macrophages. J Virol.

[B9] Hanley TM, Puryear WB, Gummuluru S, Viglianti GA (2010). PPARgamma and LXR signaling inhibit dendritic cell-mediated HIV-1 capture and trans-infection. PLoS Pathog.

[B10] Healan-Greenberg C, Waring JF, Kempf DJ, Blomme EAG, Tirona RG, Kim RB (2008). A human immunodeficiency virus protease inhibitor is a novel functional inhibitor of human pregnane X receptor. Drug Metab Dispos.

[B11] Jarrin I, Geskus R, Bhaskaran K, Prins M, Perez-Hoyos S, Muga R (2008). Gender differences in HIV progression to AIDS and death in industrialized countries: slower disease progression following HIV seroconversion in women. Am J Epidemiol.

[B12] Kaur G, Mehra N (2009). Genetic determinants of HIV-1 infection and progression to AIDS: susceptibility to HIV infection. Tissue Antigens.

[B13] Kong Q, Han Z, Wei H, Huang W (2016). Co-expression of pregnane X receptor and ATP-binding cassette sub-family B member 1 in peripheral blood: a prospective indicator for drug resistance prediction in non-small cell lung cancer. Oncol Lett.

[B14] Lahiri DK, Nurnberger JIJ (1991). A rapid non-enzymatic method for the preparation of HMW DNA from blood for RFLP studies. Nucleic Acids Res.

[B15] Martínez A, Márquez A, Mendoza J, Taxonera C, Fernández-Arquero M, Díaz-Rubio M (2007). Role of the PXR gene locus in inflammatory bowel diseases. Inflamm Bowel Dis.

[B16] McLaren PJ, Fellay J (2015). Human genetic variation in HIV disease: beyond genome-wide association studies. Curr Opin HIV AIDS.

[B17] Moore LB, Parks DJ, Jones SA, Bledsoe RK, Consler TG, Stimmel JB (2000). Orphan nuclear receptors constitutive androstane receptor and pregnane X receptor share xenobiotic and steroid ligands. J Biol Chem.

[B18] Nakayama E, Meyer L, Iwamoto A, Persoz A, Nagai Y, Rouzioux C (2002). Protective effect of interleukin-4 -589T polymorphism on human immunodeficiency virus type 1 disease progression: relationship with virus load. J Infect Dis.

[B19] NP - National Programme for STD and AIDS (2012). AIDS Epidemic Update, 2010.

[B20] Okulicz JF, Marconi VC, Landrum ML, Wegner S, Weintrob A, Ganesan A (2009). Clinical outcomes of elite controllers, viremic controllers, and long-term nonprogressors in the US Department of Defense HIV natural history study. J Infect Dis.

[B21] Pena SD, Di Pietro G, Fuchshuber-Moraes M, Genro JP, Hutz MH, Kehdy F (2011). The genomic ancestry of individuals from different geographical regions of Brazil is more uniform than expected. PLoS ONE.

[B22] Ribeiro MCSA, Barata RB, Furquim MA (2006). Sociodemographic profile and utilization patterns of the public health care system (SUS) - PNAD 2003. Cien Saude Coletiva.

[B23] Schipani A, Siccardi M, D’Avolio A, Baietto L, Simiele M, Bonora S (2010). Population pharmacokinetic modeling of the association between 63396C->T pregnane X receptor polymorphism and unboosted atazanavir clearance. Antimicrob Agents Chemother.

[B24] Schote AB, Turner JD, Schiltz J, Muller CP (2007). Nuclear receptors in human immune cells: expression and correlations. Mol Immunol.

[B25] Sinxadi PZ, Leger PD, McIlleron HM, Smith PJ, Dave JA, Levitt NS (2015). Pharmacogenetics of plasma efavirenz exposure in HIV-infected adults and children in South Africa. Br J Clin Pharmacol.

[B26] Svärd J, Spiers JP, Mulcahy F, Hennessy M (2010). Nuclear receptor-mediated induction of CYP450 by antiretrovirals: functional consequences of NR1I2 (PXR) polymorphisms and differential prevalence in whites and sub-Saharan Africans. J Acquir Immune Defic Syndr.

[B27] Thorsteinsson K, Ladelund S, Jensen-Fangel S, Larsen MV, Johansen IS, Katzenstein TL (2012). Impact of gender on the risk of AIDS-defining illnesses and mortality in Danish HIV-1-infected patients: a nationwide cohort study. Scand J Infect Dis.

[B28] Urquhart BL, Tirona RG, Kim RB (2007). Nuclear receptors and the regulation of drug-metabolizing enzymes and drug transporters: implications for interindividual variability in response to drugs. J Clin Pharmacol.

[B29] Wahli W (2008). A gut feeling of the PXR, PPAR and NF-kappaB connection. J Intern Med.

[B30] Xie W, Evans RM (2001). Orphan nuclear receptors: the exotics of xenobiotics. J Biol Chem.

[B31] Zhou C (2016). Novel functions of PXR in cardiometabolic disease. Biochim Biophys Acta.

